# Hemoglobin A1C: Intracellular Heterogeneity and Functional Implications in Prediabetic and T2 Diabetic Erythrocytes

**DOI:** 10.3390/ijms26209890

**Published:** 2025-10-11

**Authors:** Galina Petukhova, Areen Wani, Gregory Barshtein, Anna Bogdanova, Ariel Koren, Carina Levin, Leonid Livshits

**Affiliations:** 1 MIGAL Galilee Research Institute, Kiryat Shmona 11016, Israel; galina.pe@migal.org.il (G.P.); areen.wa@migal.org.il (A.W.); 2 Biochemistry Department, The Faculty of Medicine, The Hebrew University of Jerusalem, Jerusalem 9112102, Israel; gregoryba@ekmd.huji.ac.il; 3 Red Blood Cell Research Group, Vetsuisse Faculty, Institute of Veterinary Physiology, University of Zurich, 8057 Zürich, Switzerland; annab@access.uzh.ch; 4 The Zurich Center for Integrative Human Physiology (ZIHP), 8057 Zürich, Switzerland; 5 Pediatric Hematology Unit, Emek Medical Center, Afula 1834111, Israel; korenariel48@gmail.com (A.K.); levin_c@clalit.org.il (C.L.); 6 The Bruce and Ruth Rapaport Faculty of Medicine, Technion—Israel Institute of Technology, Haifa 3200003, Israel; 7 Faculty of Sciences, Tel Hai Academic College, Upper Galilee, Kiryat Shmona 12208, Israel

**Keywords:** hemoglobin A1C, hemoglobin distribution, red blood cells, prediabetes, T2 diabetes, calcium

## Abstract

Hemoglobin A1C (HbA1C), a non-enzymatically glycated form of adult hemoglobin (HbA0), is a widely used biomarker for diabetes. Its concentration is strongly correlated with the long-term glycemic state and the risk of diabetes development. However, beyond its diagnostic role, its physiological functions remain poorly understood. To fill this gap, we investigated the intracellular distribution of HbA1C and its potential impact on red blood cell (RBC) functions. Specifically, the differences in cytosolic and membrane pools of HbA1C in RBCs from individuals with prediabetes, overt type 2 diabetes (T2D), and healthy controls were explored. Our cross-sectional findings confirmed the intracellular heterogeneity of HbA1C and revealed a strong correlation between fluctuations in HbA1C and those of other hemoglobin isoforms, specifically HbA2 and HbA0. This correlation was particularly evident in the context of diabetes or acute exposure to Ca^2+^-depleted environments. We also observed that short-term hyperglycemia does not significantly alter HbA1C intracellular localization. Furthermore, we found that the intracellular distribution of HbA1C is correlated with several physiological properties of RBCs, with these links varying according to the specific pathological abnormalities associated with pre- and overt diabetes. Further research is required to fully understand the mechanisms and implications of these observations.

## 1. Introduction

Type 2 (non-insulin dependent) diabetes mellitus (T2D) is a complex metabolic disorder affecting nearly half a billion people worldwide. It is characterized by hyperglycemia resulting from a combination of insulin resistance and inadequate insulin secretion by pancreatic beta cells [1]. This chronic condition not only imposes significant health burdens but also increases the risk of long-term complications, including nephropathy, retinopathy, neuropathy, cardiovascular disease, peripheral vascular disease, dental problems, and sexual dysfunction [2,3,4,5].

Prediabetes is a significant risk condition for T2D development. Individuals with impaired fasting glucose, impaired glucose tolerance, elevated levels of glycated hemoglobin (HbA1C)—between 5.7% and 6.4%—or a history of gestational diabetes fall into this category [6]. Globally, the number of adults with prediabetes is estimated to be around 400 million, with a rapid increase observed annually [7]. Individuals with prediabetes are at increased risk of developing both overt diabetes and cardiovascular disease. It is crucial to understand that prediabetes is a diverse condition; it can lead to rapid diabetes onset in some individuals, whereas others may stay in the prediabetic stage for a long time.

HbA1C, a non-enzymatically glycated form of major adult hemoglobin (HbA0), is of significant clinical interest. This modified hemoglobin (Hb) forms through a series of chemical reactions of glucose with the N-terminal amino group of the HbA0 β-chain [8]. It accounts for 80% of total glycated Hb; the remaining glycohemoglobins have glucose, glucose-6-phosphate, fructose-1-6-diphosphate, or pyruvic acid bound to ε-amino groups of lysine residues or the N terminus of the α-chain. HbA1C is more susceptible to oxidation and degradation than non-glycated Hb [9,10], contributing to oxidative stress by increasing the release of heme and free iron in association with free radicals [11,12,13,14]. Moreover, its negative charge exceeds that of the HbA0 variant, enabling easy detection of HbA1C using techniques such as HPLC [15]. However, although HbA1C is widely used as a diagnostic marker for diabetes [16,17], its physiological functions remain mostly unexamined.

To our knowledge, the intracellular distribution of HbA1C and the potential impact of Hb glycation on its localization have never been studied. We recently explored the distribution of adult Hb variants HbA0 and HbA2 between cytosolic and membrane fractions, and their potential selective localization under plasma-borne stress [18]. We observed preferential localization of HbA2 in the pre-membrane pool (5–9% of total Hb on the membrane vs. ~3% in intact red blood cells [RBCs] and cytosol), with a concurrent decrease in pre-membrane HbA0. Moreover, we identified a key role for both extracellular and intracellular Ca^2+^ and the potential involvement of anion exchanger 1 (AE1, also known as band 3 protein) in regulating the distribution of these Hb isoforms between the cytosol and the membrane. Considering the direct interrelationships between Ca^2+^, AE1, and membrane-associated Hb [19,20,21,22,23,24,25,26,27,28], we hypothesized that interaction of various Hb variants with the membrane may affect the RBCs’ morphology, redox state, rheology, membrane permeability, and glucose metabolism. This hypothesis motivated us to explore the subcellular localization preference of HbA1C, and its possible involvement in RBC physiology and rheological pathways. Furthermore, since HbA1C levels are relatively low in healthy individuals (<5.7%) and their elevation is caused by metabolic disturbances, we expanded our cross-sectional study to examine the corresponding features and pathways in erythrocytes obtained from individuals with prediabetes and overt T2D.

## 2. Results

**Figure 1:**
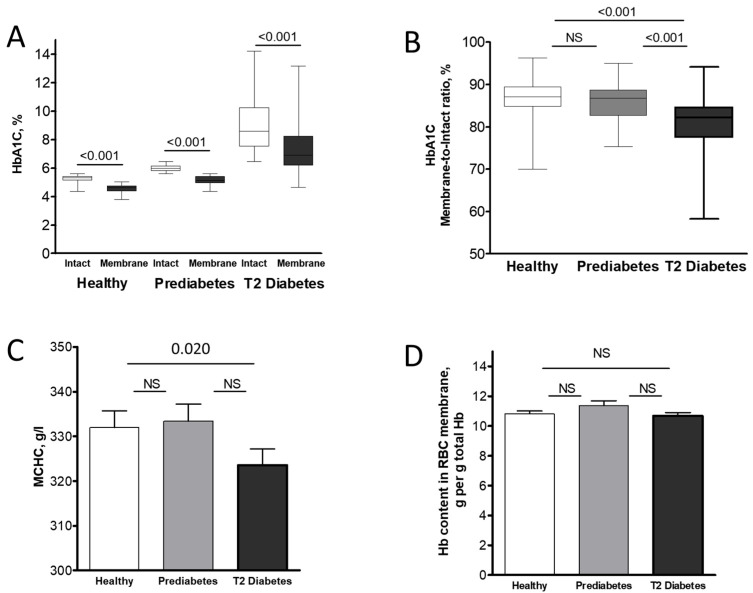
Subcellular distribution of HbA1C in healthy, prediabetic, and T2 diabetic individuals. The percentage of HbA1C relative to total hemoglobin (**A**) and membrane-to-intact compartment HbA1C ratios (**B**) for healthy (*n* = 56), prediabetic (*n* = 53), and T2 diabetic (*n* = 56) individuals are shown. Blood samples collected into heparin-supplemented tubes were kept at room temperature for no more than 4 h before measurement. RBCs were isolated from plasma and buffy coat by brief centrifugation at 1700× *g*, and Hb isoform measurements were immediately performed on intact RBCs; RBC membranes were isolated as described in Section 4.4. Wilcoxon signed-rank test was used to assess significance for the membrane and intact fractions, and the ratios between them, within each group; data are presented as median ± CI. Significance level: *p* < 0.05; NS, non-significant. Hb concentration in intact RBCs (**C**) and the corresponding Hb content in their membrane fractions (**D**) were determined using Drabkin’s reagent as described in Section 4.4. The data were found to be normally distributed and were compared using paired Student’s *t*-test (all *p* > 0.05).

Demographic and RBC data for the study participants are summarized in Table 1. Comparison of the intact and near-membrane fractions of HbA1C (Figure 1A) and other Hb isoforms (Table 2) in RBCs from healthy, prediabetic, and overt T2 diabetic individuals revealed significant isoform-dependent differences. Given the minimal variations in Hb isoform distribution between intact RBCs and their cytosol (Table S1), we considered the isoform ratios in intact RBCs to reflect those in the cytosolic compartment. Similar to HbA0 and in contrast to the membrane-enriched HbA2, the near-membrane fraction of HbA1C was lower at the membrane compared to the intact (cytosolic) portions across all examined individuals. Interestingly, the rise in intact HbA1C, which indicates prolonged exposure to high glucose levels in prediabetic and T2 diabetic individuals, was mirrored in the HbA1C levels in the RBC membranes of these patients (Figure 1B). Variations in intracellular Hb content and concentrations, specifically mean corpuscular hemoglobin (MCH) and its concentration (MCHC) (as detailed in Table 1 and Figure 1C), along with Hb concentration in the membrane fractions (Figure 1D), enable associating observed changes in the percentage of HbA1C and other Hb isoforms relative to total Hb with the quantitative abundance of these isoforms in different RBC compartments (Table S2).

To examine the relationship between the subcellular distribution of Hb variants and HbA1C, we pooled the data for intact (cytosolic) and membrane distributions of Hb isoforms across all cohorts and correlated their variation with HbA1C by calculating Pearson’s correlation coefficients (Table 3). This analysis revealed significant positive correlations for HbA0 and negative correlations for HbA2 in both intact RBCs and their membrane fractions. Correlations with HbF were non-significant. Analyzing correlations within individual groups, HbA1C was found to correlate with HbA0 (positively) and HbA2 (inversely) in both the cytosolic and membrane pools only in diabetic RBCs. The other cohorts exhibited significant correlations involving HbA1C and HbA0 or HbA2 only within the membrane fraction. No correlations were observed between HbA1C and HbF in any of the examined groups.

**Figure 2:**
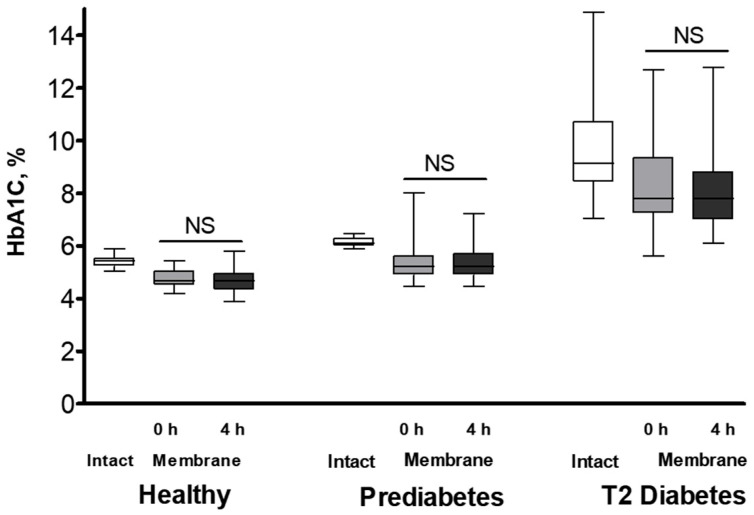
Changes in HbA1C concentration in intact RBCs and their membrane compartments following a 4 h incubation under hyperglycemic conditions. RBCs from healthy (*n* = 18), prediabetic (*n* = 17), and T2 diabetic (*n* = 17) individuals were incubated in plasma-mimicking buffer (PMB) supplemented with 10 mM glucose. The percentage of HbA1C relative to total Hb was determined in both intact cells and isolated membrane fractions before and after the 4 h incubation period. Statistical significance of changes within each group (baseline vs. 4 h) for RBC membrane data was determined using the Wilcoxon signed-rank test. Significance level: *p* < 0.05; NS, non-significant.

We then investigated the temporal dynamics of HbA1c and other Hb variants’ cellular redistribution by incubating RBCs in plasma-mimicking buffer (PMB) with a near-physiological hyperglycemic glucose concentration (10 mM) for 4 h. A comparison of the membrane distribution of Hb isoforms before and after incubation (Figure 2 and Table S3) showed only minimal changes in the near-membrane content of each isoform across all groups. Consistently, these minor isoform-specific changes did not result in significant alterations to the total Hb membrane concentration in any of the studied groups (Figure S1).

**Figure 3:**
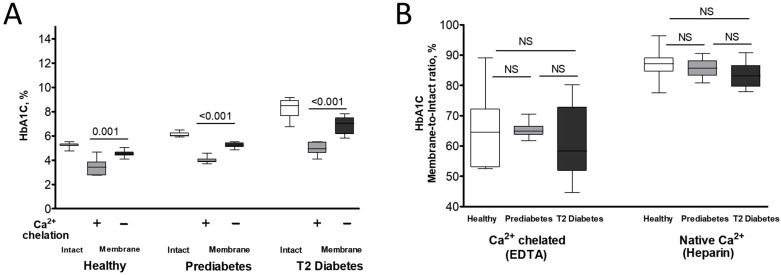
Effect of Ca^2+^ chelation on HbA1C subcellular distribution in healthy (*n* = 10), prediabetic (*n* = 9), and T2 diabetic (*n* = 8) individuals. The percentage of HbA1C relative to total Hb (**A**) and membrane-to-intact HbA1C ratios (**B**) in RBCs, which were collected in parallel into K_3_EDTA- or heparin-supplemented tubes, are displayed. RBCs were isolated and measured as described in Material and Method section. Significance was determined by comparing the corresponding RBC membrane datasets within each group using Wilcoxon signed-rank test. Significance level: *p* < 0.05; NS, non-significant.

To explore whether HbA1C intracellular distribution is regulated by extracellular Ca^2+^, we collected erythrocytes in tubes supplemented with a Ca^2+^ chelator, K_3_EDTA, in parallel to their collection in tubes with heparin anticoagulant. Ca^2+^ chelation led to decreased membrane levels of HbA1C compared to control (heparin-preserved) RBCs (Figure 3A). This response was similar to the intracellular redistribution of HbA0 along with an increase in the membrane association of HbA2 (Table S4). Interestingly, similar to the cells exposed to physiological Ca^2+^ levels (in heparin tubes), no substantial variations were found in the HbA1C membrane-to-intact ratios among EDTA-treated RBCs from healthy, prediabetic, and diabetic individuals (Figure 3B).

To further explore the relationship between HbA1C and RBC function, we examined the correlation between HbA1C and various RBC properties in healthy individuals, as well as in those with prediabetes and diabetes. Specifically, K^+^ loss (a marker of membrane permeability), median elongation rate (RBC deformability), glucose consumption, lactate efflux rate (RBC metabolism), and intracellular levels of reduced thiols (redox state) vs. HbA1C levels in intact cells and in pre-membrane Hb fraction were analyzed (Table 4). The raw data and a statistical comparison of these parameters in healthy, prediabetic, and overt T2 diabetic RBCs are presented in Table S5. We found that the fraction of intact HbA1C was specifically correlated with lactate efflux rate (inversely) and reduced thiol content (positively) in healthy RBCs, and with mean elongation rate (inversely) in prediabetic erythrocytes. The membrane-bound HbA1C pool was negatively correlated with glucose consumption rate in prediabetic RBCs. These observed correlations imply a possible relationship between HbA1C and the physiological regulation of metabolic processes, highlighting abnormalities in these processes within diabetic conditions.

## 3. Discussion

In light of the global diabetes epidemic [29], HbA1C has become one of the most important biomarkers in clinical practice in the last few decades. This study aimed to clarify specific questions regarding the cellular properties of this form of Hb. The present study reveals three major findings. First, the distribution of HbA1C is heterogeneous, with a lower concentration at the RBC membrane than in the cytosolic compartment. This ratio remained consistent, regardless of overall HbA1C levels, in all tested groups of study participants. Second, there was a correlation between changes in HbA1C level and those of other Hb isoforms, specifically HbA2. Third, our findings suggest that HbA1C intracellular levels correlate with certain specific metabolic and rheological RBC properties, and these effects may vary depending on diabetes-related changes in the distribution of HbA1C between the intact cell and the membrane compartments.

### 3.1. Heterogeneous Intracellular Distribution of HbA1C and Interference with Other Hb Isoforms

The proportion of HbA1C in the pre-membrane pool was significantly lower than in the cytosolic compartment (Figure 1A). Interestingly, our recent findings ([18]; see also Table S1) revealed a significantly higher proportion of the HbA2 isoform in the pre-membrane pool compared to intact RBCs and the cytosol. This raises the question of whether particular features of the Hb molecules are used for the sorting of particular isoforms at the membrane. One such feature might be Hb charge. Positively charged HbA2 [30] seems to favor binding to the membrane, whereas negatively charged HbA1C [15] has a lower probability of joining the pre-membrane Hb pool. The total amount of all Hb variants in the pre-membrane pool remained constant in healthy, prediabetic, and T2 diabetic individuals (Figure 1D), suggesting a constant number of “binding sites” for all Hb molecules at the membrane. Different isoforms of Hb seem to compete for these binding sites as the fractions of Hb isoforms are inversely related, as observed in the RBC membranes of all experimental groups (Table 3).

As already noted, N-terminal cytosolic domain of AE1 enriched with anionic amino acids, is widely recognized as a primary membrane binding site for Hb [24,25,26,27,28]. This negatively charged domain docks within the central cavity formed by the four globins of Hb, which is positively charged. This cavity opens wider as Hb deoxygenates, making the interaction O_2_-dependent. Notably, HbA2 exhibits a high affinity for AE1, with its membrane association not being solely dependent on electrostatic interactions [31]. Considering that a single RBC typically contains around 260 million Hb molecules [32], our data reveal approximately 507,000 HbA2 and 585,000 HbA1C molecules in the pre-membrane fraction of erythrocytes from healthy individuals. Given AE1’s overwhelming presence (~1.2 × 10^6^ copies per cell), it logically represents a principal target for interaction with various Hb isoforms, but not the only one, as oxygenated Hb is less prone to dock to the cytosolic domain of AE1.

Hb has also been reported to interact with spectrin [33], as well as with anionic phosphatidylserine [34,35]. To the best of our knowledge, no data have been reported regarding a preferential interaction of HbA1C with any specific membrane component. Moreover, the modifications that convert HbA0 to HbA1C could potentially influence its binding properties. However, these reasons may provide an explanation for the small, but significant decrease in the membrane vs. intact proportion of HbA1C, especially in T2 diabetic individuals (Figure 1B). Given the significant alterations observed in the RBC membrane proteome in patients with T2D and impaired glucose tolerance [36,37], identifying the specific membrane targets of HbA1C, both those that are unique and those that are shared with other Hb isoforms, is crucial.

Although all samples were oxygenated prior to their examination, the potential contribution of Hb oxygenation should also be considered. As mentioned above, the mechanisms governing AE1’s interaction with Hb, in either oxygenated or deoxygenated form, are well described [25,28,38,39,40,41]. HbA1C exhibits an approximately 10-fold higher affinity to oxygen than the non-glycosylated form of HbA0 [42]. Therefore, we might expect a lower prevalence of HbA1C in the pre-membrane fraction compared to HbA0 (a hypothesis supported by our data). If this holds true, O_2_ release occurs first from the pre-membrane Hb fraction enriched with HbA0 and A2 with lower average oxygen affinity, followed by O_2_ release by the cytosolic Hb fraction enriched with HbA1C. This further suggests that oxygenation of HbA1C occurs at a much lower partial pressure of oxygen than for HbA0 or HbA2, reducing its probability of docking at the cytosolic domain of AE1 with a higher affinity for the deoxygenated Hb state when in the circulation. Oxygen affinity of HbA2 is comparable to that of HbA0 [31]; and the preferential binding of HbA2 to the cytosolic domain of AE1 compared to HbA0 is governed by other factors, including a positive surface charge.

Taken together, the obtained data suggest the following order of preference for binding to the membrane for the Hb isoforms: HbA2 > HbA0 > HbA1C, which reflects the differences in Hb isoform distribution between the cytosol and the pre-membrane pool (Table 2). This uneven distribution of Hb isoforms at the membrane defines functional differences between the pre-membrane and bulk cytosolic Hb; the physiological role remains to be explored. We acknowledge that direct experimental validation of this hypothesis is still lacking, warranting further studies.

**Figure 4:**
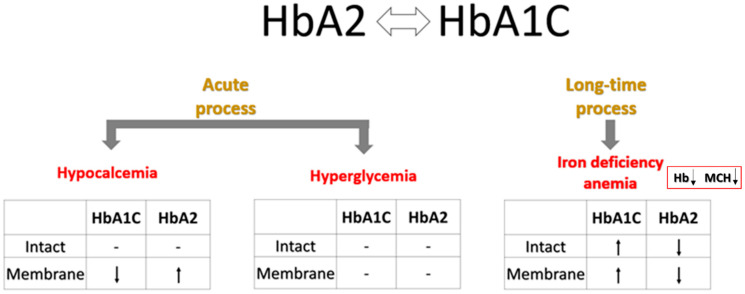
Proposed mechanisms of reciprocal influence between HbA1C and HbA2 isoforms on their intracellular levels and localization.

In contrast to the inverse relationship between HbA1C and HbA2 contents observed in the membrane fractions of all examined groups, bulk levels of these Hb variants showed an opposite correlation exclusively in the RBCs of subjects with overt T2D. This phenomenon may be explained by several mechanisms that become prominent during the development of diabetic pathogenesis (Figure 4). A more chronic mechanism, particularly associated with overt diabetes, could be the onset of iron deficiency anemia (IDA). T2D and iron deficiency share a recognized and complex bidirectional relationship. IDA is notably more prevalent in individuals with T2D, with reported rates ranging from 13% to 47% across various studies [43]. This increased incidence of IDA in diabetic patients is driven by several interconnected mechanisms, such as diminished erythropoiesis due to decreased erythropoietin production in diabetic nephropathy [44]. The chronic inflammation characteristic of T2D elevates the level of hepcidin, a key regulator of iron homeostasis, impairing intestinal iron absorption and restricting the release of stored iron, thus promoting iron deficiency [45,46]. In addition, while not a direct cause of IDA, metformin, a common T2D medication, has been linked to vitamin B12 deficiency, which can contribute to or exacerbate anemia [47,48]. Finally, dietary modifications or altered eating habits in individuals with T2D may lead to insufficient iron intake [49]. Consequently, IDA commonly presents with a decrease in HbA2 [50], likely due to the more pronounced impact of iron deficiency on δ-globin synthesis and potentially reduced α-globin availability. Consistent with this, our study also revealed a decline in intact HbA2 within the T2 diabetic cohort samples (Table 2).

Variations in blood Ca^2+^ levels are a well-documented phenomenon in patients with T2D [51]. Specifically, several studies have reported elevated Ca^2+^ concentrations in individuals with T2D compared to healthy controls [52,53]. Moreover, a potential association between increased Ca^2+^ levels in the blood and a higher risk of developing diabetes has also been suggested [54]. To address these abnormalities, EDTA-based Ca^2+^-chelation therapies, such as the recent Trial to Assess Chelation Therapy (TACT) project, have been proposed as a means of preventing side-effect complications in diabetes treatment [55]. Intriguingly, our findings (Figure 3B and Table S4) demonstrate a significant decrease in HbA1C and an increase in HbA2 fractions in the total membrane Hb pool following EDTA treatment. Consequently, we can suggest that EDTA changes the influence of HbA1C on RBC mechanisms regulated by membrane components. Our prior work [18] established a direct link between plasma Ca^2+^ depletion by EDTA, Hb relocation within the RBC, and alterations in erythrocyte membrane permeability and metabolic properties. The current study suggests that some of these mechanisms are connected to, and potentially regulated by, HbA1C (Table 4, and see Section 3.2). This hypothesis warrants further investigation to understand clinical outcomes associated with RBC transfusion (where Ca^2+^-free storage solutions are routinely used) [56] and hypocalcemia-related complications [57,58,59,60,61,62].

In contrast, acute hyperglycemia did not significantly alter the intracellular distribution of HbA1C (or other Hb isoforms) (Figure 2). Erythrocytes exhibit a substantial metabolic requirement for glucose [63], which enters the cells via insulin-independent glucose transporter 1 (GLUT1), the primary glucose carrier in human RBCs [64]. Glucose binding and transport induce significant conformational changes in GLUT1 [65,66,67,68]. Given the high abundance of GLUT1 in the RBC membrane, these dynamic structural changes likely contribute to the membrane’s overall organization and function [67]. Our findings of a modest effect of acute hyperglycemia on HbA1C’s intracellular arrangement present several interesting perspectives. Our data suggest the lack of direct interaction between GLUT1 and any Hb isoform. However, the lack of observed effects of glucose influx and/or its utilization on HbA1C redistribution does not rule out a potential influence of HbA1C on these processes. Investigating this possibility warrants a separate future study.

### 3.2. Possible Involvement of HbA1C in RBC Physiology and Rheology

Diabetes and its associated complications are characterized by hyperglycemic toxicity [69]. However, hyperglycemia is often a relatively late biomarker of diabetes. In many patients, insulin levels initially rise to compensate for increased insulin resistance. When the demand for insulin exceeds its production, glucose levels rise. In other words, before being exposed to high glucose concentrations (leading to increased HbA1C), RBCs are exposed to numerous non-hyperglycemic diabetic stimuli for a relatively extended period. This may explain why most of the examined features in both prediabetic and diabetic RBCs are poorly correlated with HbA1C levels (Table 4). In addition, a correlative approach can be useful to identify the physiological roles of HbA1C. This can be particularly useful when studying RBCs from prediabetic individuals, where the influence of hyperglycemia is still mild. Correlations between HbA1C and specific physiological features in non-hyperglycemic RBCs may also provide evidence for the possible regulatory function of HbA1C in the organism.

As demonstrated previously [70,71] and confirmed in the present study (Table 4), the rate of glucose uptake in diabetic erythrocytes is significantly reduced compared to that in healthy individuals. There were no significant changes in GLUT1 abundance in RBCs of healthy vs. diabetic individuals; however, increased glucose affinity of GLUT1 and structural alterations in the transporter [71], particularly in the outer domain [72], have been proposed as potential mechanisms for this reduced glucose uptake. Intriguingly, we observed an inverse correlation between the fraction of membrane-bound HbA1C and glucose-uptake rate only in prediabetic patients, but not in healthy or diabetic individuals. However, when analyzing the correlation between the intact HbA1C fraction and glucose-uptake rate across the combined cohort of healthy and T2 diabetic individuals, we found a significant inverse linear relationship between these parameters (*p* = 0.009). This result is similar to the findings of Porter-Turner and colleagues [71]. Despite similar trends in individual groups, overall significance for the total cohort was lacking. We hypothesize that this discrepancy may arise from different pathological stimuli affecting glucose-uptake and consumption rates in pre- and overt diabetic individuals; we will explore the nature of these stimuli in future studies.

Among the studied features, we found a correlation between intact HbA1C levels and lactate release in healthy erythrocytes. Lactate is an end-product of glycolysis, a key metabolic pathway that utilizes glucose to provide energy. Therefore, lactate production is directly linked to glucose uptake and glycolytic rate [63,73]. In contrast to the observed correlation for lactate release, we did not observe a corresponding correlation between HbA1C content and the rate of glucose consumption in healthy subjects, optionally suggesting that HbA1C primarily influences lactate efflux rather than production. To date, three primary pathways for lactate transport have been identified: (a) the H^+^-monocarboxylate transporter (MCT) pathway, (b) AE1-mediated exchange with inorganic anions, and (c) passive diffusion across the lipid bilayer (see more details in Ref. [74]). In human RBCs, the MCT pathway—specifically via the MCT1 transporter—is the predominant pathway for lactate exchange [75]. In general, regulation of all three pathways may potentially involve HbA1C, but the precise mechanisms underlying this link require further investigation. Moreover, the lack of correlation between HbA1C and lactate release in prediabetic and diabetic participants may relate to metabolic alterations in the native mechanism; therefore, further study may enhance clinical significance.

A positive correlation of the intact HbA1C pool with intracellular deprotonated reduced thiol (thiolate) content was revealed in healthy RBCs. Thiolate anions, formed by the deprotonation of sulfhydryl groups (R-SH), are present in proteins with free cysteine residues (e.g., Hb) and small substances such as glutathione (GSH). Previous studies have reported a negative correlation between HbA1C and thiol content in both healthy and diabetic individuals [76], suggesting that thiol groups may be targets of glycation [77,78]. Decreased intracellular GSH levels are well-documented in patients with T2D and diabetic complications (see literature summary in Ref. [79]). Unfortunately, the VARIANT™ II TURBO Hemoglobin Testing System does not allow measuring the Hb- or specifically HbA1C fractions directly interacting with GSH in contrast, for example, to the method by Al-Abed et al. (2001) [80]. In the current study, we specifically evaluated intracellular levels of reduced thiols using the fluorescent dye monobromobimane (MBBR), and found a positive correlation with HbA1C levels. It is important to point out that the content of thiolate anions and their reactivity with MBBR are strongly pH-dependent [81]. In light of the previously confirmed positive correlation between intracellular pH and HbA1C levels [82], further investigation into the interplay of pH, thiol and HbA1C concentrations holds significant potential for future insights.

RBC deformability, a crucial hemodynamic property, enables the cells to dynamically alter their shape in response to flow conditions, thereby minimizing vascular resistance. This adaptability is essential for navigating narrow capillaries and the splenic vasculature, preventing their sequestration and premature clearance [83,84]. Reduced deformability impairs perfusion and oxygen delivery to peripheral tissues [85,86]. Notably, interactions between Hb and the RBC membrane have been implicated in compromised deformability [87], with recent evidence suggesting that deoxyHb-binding contributes to this reduction [88]. Our recent findings further indicate a correlation between decreased RBC deformability and the altered distribution of specific oxygenated Hb isoforms, particularly HbA2 and HbA0, between the cytosol and the cell membrane [26]. Decreased RBC deformability is a well-established characteristic in T2 diabetic patients [89,90,91,92,93,94]. However, despite significant research efforts to define a precise HbA1C threshold for the onset of impaired RBC deformability (e.g., Ref. [95]), the underlying mechanisms linking HbA1C to this alteration remain unclear. Surprisingly, in our study, we did not observe significant differences in mean elongation rate values between the studied groups. Importantly, all groups were well-matched and did not exhibit substantial differences in age or other examined RBC indices, except for cellular and membrane HbA1C levels. A significant correlation was found between deformability and HbA1C levels only in prediabetic RBCs (Table 4). Moreover, the overall correlation between HbA1C and mean elongation rate was minimal, suggesting a direct link between early diabetic events occurring in normoglycemia and changes in deformability. As a possible scenario, the contribution of hyperinsulinemia, which is associated with the developed insulin resistance in early diabetic pathogenesis [96,97,98], may be considered. Several previous studies [99,100] have linked metabolic abnormalities associated with insulin resistance to negative alterations in blood rheology. However, the absence of plasma insulin measurements in our study precludes confirmation of the hypothesized hyperinsulinemic nature of the observed correlations.

### 3.3. Limitations

This study has several limitations that should be carefully addressed in future research. First, its cross-sectional design restricts our ability to perform a longitudinal analysis of the observed correlations, which is especially important for understanding how diabetes progresses and how complications develop over time. Second, a major constraint is our reliance on leftover blood samples, which were selected solely based on basic factors such as age and HbA1C levels. We lacked access to or control over other influential factors, such as detailed medical histories, lifestyle, treatment plans, or additional laboratory data unrelated to blood. Future studies will aim to overcome these limitations by designing dedicated research to rigorously test each hypothesis generated in this work.

## 4. Materials and Methods

### 4.1. Blood Samples

Residual adult blood samples collected in heparin sulfate- or K_3_EDTA-supplemented tubes for routine clinical analysis at the central laboratory of Emek Medical Center (EMC) in Afula (Israel) between 2022 and 2025 were randomly selected by participant’s age (>20 years), and HbA1C levels of <5.7% (defined as healthy individuals), 5.7–6.4% (prediabetes), and >6.5% (T2D). Blood samples collected into heparin-supplemented tubes were kept at room temperature prior to the experimental manipulations. Total time elapsed between blood collection and measurement did not exceed 4 h. The study was conducted following the Declaration of Helsinki and approved by the EMC ethics committee (EMC-0085-21).

### 4.2. Buffers and Chemicals

Plasma-mimicking buffer (PMB) contained 140 mM NaCl, 4 mM KCl, 0.75 mM MgSO_4_, 10 mM glucose, 0.015 mM ZnCl_2_, 0.2 mM glycine, 0.2 mM sodium glutamate, 0.2 mM alanine, 0.1 mM arginine, 0.6 mM glutamine, and 20 mM HEPES, adjusted to pH 7.4 with imidazole, and then supplemented with 0.01% *w*/*v* bovine serum albumin and 2 mM CaCl_2_. These and other chemicals were purchased from Sigma-Aldrich Israel (Rehovot, Israel), unless otherwise specified.

### 4.3. Hemoglobin Variant Analysis

**Figure 5:**
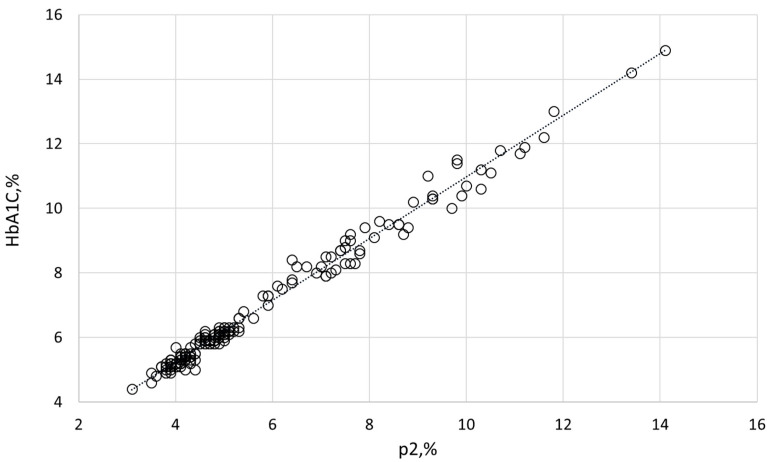
Correlation between p2, measured by the VARIANT™ II TURBO Hemoglobin Testing System, and the routine clinical index HbA1C, measured by the D-100 Hemoglobin Testing System.

HbA1C and other Hb variants were quantified by HPLC. The VARIANT™ II TURBO Hemoglobin Testing System (Bio-Rad, Hercules, CA, USA) was employed to separate Hb variants by cation-exchange chromatography in a salt gradient. Calibrations and controls were provided by the manufacturer with each batch. Samples were analyzed using the VARIANT™ II β-thalassemia Short Program. The analysis involved monitoring retention times, area percentages, and concentrations of various peaks and windows corresponding to different Hb variants: HbF (retention time of 1.1 min, 0.98–1.2 min window), HbA0 (2.5 min, 2.0–3.0 min), HbA2 (3.65 min, 3.57–3.75 min), and p2 (related to HbA1C; 1.39 min, 1.28–1.5 min). The measured p2 values exhibited a strong correlation (*p* = 0.9932) with the corresponding, clinically accepted HbA1C index (determined by the D-100 Hemoglobin Testing System; Bio-Rad), as shown in Figure 5. The intact and membrane HbA1C values presented in this study were recalculated from the corresponding p2 values.

### 4.4. RBC Membrane Preparation

The membrane fraction of RBCs was isolated following a previously described protocol with minimal modifications [18]. Briefly, RBCs were isolated from plasma and buffy coat by short (5 min) centrifugation at 1700× *g* at room temperature. Then, a 150 µL aliquot of RBCs was incubated in 20 volumes of ice-cold HEPES-based hypoosmotic solution (20 mM HEPES/NaOH, 1 mM PMSF, pH 7.4) for 10 min, followed by centrifugation at 4 °C for 15 min at 14,000× *g*. This procedure was repeated three times before proceeding with measurements of Hb isoform distribution or other cellular features. The Hb content was determined as cyanmethemoglobin using Drabkin’s reagent according to the manufacturer’s protocol.

### 4.5. Glucose Consumption, Lactate Release, and Potassium (K^+^)-Leakage Studies

The tests were performed using a GEM^®^ Premier™ 5000 blood gas analyzer (Werfen, Bedford, MA, USA) according to our previously published protocol [18]. Briefly, after removal of the plasma and the buffy coat as described above, RBCs were quickly washed three times with PMB. Then, the cells were centrifuged at 1700× *g* for 5 min, the supernatant was discarded, and the cells were resuspended in a fresh medium. The cells were quickly mixed, and basal levels of extracellular K^+^, glucose, and lactate were immediately detected using the blood gas analyzer. The cells were then incubated for 4 h at 37 °C in a shaker, and the measurements of extracellular K^+^, glucose, and lactate levels in PMB were repeated. Changes in K^+^, glucose, and lactate concentrations in PMB, reflecting K^+^ loss and glucose conversion to lactate, over 4 h, were expressed as millimoles per gram of Hb per hour. To correlate these changes with alterations in intact and membrane HbA1C and other Hb variants, RBC samples were collected and analyzed at both 0 and 4 h time points.

### 4.6. Determination of RBC Deformability

RBC deformability was assessed using a computerized cell flow analyzer [101,102,103]. Briefly, a 50 µL aliquot of RBC suspension (1% hematocrit, in the same medium used for pretreatment) was applied to an uncoated glass slide in a flow chamber. After a 10 min adhesion period, buffer flow was initiated, and the deformation of adherent RBCs was monitored at a shear stress of 3 Pa. For each measurement, 15–20 randomly selected fields (0.1 mm^2^ each) were analyzed. Image analysis was used to determine the elongation ratios (ER) of individual cells and their distribution within the RBC population (ranging from 12,000 to 15,000 cells per sample). ER was calculated as the ratio of the major axis to the minor axis of each cell. An ER of 1 indicates a round, non-deformed RBC, while an ER of 3 signifies an extra- deformed, elongated erythrocyte.

### 4.7. Measurement of Deprotonated Reduced Thiols

The abundance of deprotonated reduced thiols in intact cells was measured using flow cytometry and FluoroPure MBBR (Thermo Fischer Scientific, Waltham, MA, USA). RBCs were first washed to remove plasma and buffy coat; 1 µL of packed RBCs was resuspended in 1 mL PMB supplemented with 100 µM MBBR dye. The samples were incubated for 1 h at 37 °C in the dark for thiol-labeling. Fluorescence intensity of stained RBCs was measured using a Navios EX flow cytometer (Beckman Coulter, Brea, CA, USA). Measurements were repeated at least twice, analyzing over 30,000 cells per sample. All data were analyzed using Kaluza Analysis Software (Beckman Coulter, https://www.beckman.co.il/flow-cytometry/software/kaluza).

### 4.8. Statistics

Data for the entire study were analyzed using GraphPad 5 software. The normality of distribution of the values obtained in each experimental set was evaluated by Shapiro–Wilk test, and those with *p* > 0.05 were considered normally distributed. For those parameters showing normal distribution, paired-matched values were compared by paired Student’s *t*-test. For the datasets that were not normally distributed, the Wilcoxon signed-rank test was used. For all analyses, a two-tailed test with *p* < 0.05 was accepted as statistically significant. For more details, see the figure legends.

## 5. Conclusions

This study reveals a heterogeneous distribution of HbA1c in human RBCs, specifically noting a reduced presence in the pre-membrane pool. We observed a strong correlation between fluctuations in HbA1C and other Hb isoforms, such as HbA2 and HbA0, especially under conditions of overt T2D or acute exposure to Ca^2+^-depleted environments. Notably, short-term hyperglycemia had a minimal impact on HbA1C intracellular localization. Furthermore, our findings indicate a significant correlation between HbA1C intracellular distribution and several physiological properties of RBCs. These relationships may vary based on the specific pathological abnormalities associated with pre- and overt diabetic conditions. Further research is essential to fully elucidate the underlying causes and consequences of these observed phenomena.

## Figures and Tables

**Table 1:** Red blood cell (RBC) properties and distribution of hemoglobin (Hb) isoforms in the participants. Abbreviations: M, male, F, female; RBC, red blood cell count; HCT, hematocrit; MCV, mean corpuscular volume; MCH, mean corpuscular hemoglobin; MCHC, mean corpuscular hemoglobin concentration; RDW, RBC distribution width. RBC indices were measured by Advia2120 analyzer (Siemens Healthcare Diagnostics Manufacturing Limited, Swords, Co., Dublin, Ireland) Hb variants (HbF, HbA2, HbA0, and p2) were analyzed using the VARIANT™ II TURBO Hemoglobin Testing System. HbA1C was measured with the D-100 Hemoglobin Testing System. The percentage of each Hb isoform out of the total Hb is shown. Data are presented as means ± SD. Statistical significance was determined using paired Student’s *t*-test compared to the healthy group for prediabetic individuals and compared to healthy/prediabetic groups for type 2 (T2) diabetic individuals. Significance level: *p* < 0.05; NS, non-significant.

	Reference Ranges	Healthy	Prediabetes	Type 2 (T2) Diabetes
n		56	53	56
Age (years)		58.3 ± 14.0	65.3 ± 11.3	62.6 ± 14.3
M/F		24/32	29/24	30/26
HbA1C (%)	<5.7	5.24 ± 0.26	6.01 ± 0.16	8.88 ± 1.67
RBC (10^6^/µL)	Female: 4.0–5.0 Male: 4.5–5.5	4.71 ± 0.55	4.77 ± 0.51 ^NS^	5.03 ± 0.63 ^0.005/0.021^
HCT (%)	Female: 37.0–47.0 Male: 40.0–54.0	41.1 ± 4.6	41.8 ± 3.8 ^NS^	42.6 ± 4.9 ^NS/NS^
Hb (g/dL)	Female: 12.0–15.0 Male: 14.0–17.0	13.6 ± 1.6	13.8 ± 1.2 ^NS^	13.9 ± 1.8 ^NS/NS^
MCV (fL)	Female: 80.0–94.0 Male: 80.0–95.0	87.1 ± 8.2	88.2 ± 5.8 ^NS^	84.8 ± 6.6 ^NS/0.007^
MCH (pg)	27.0–31.0	29.0 ± 2.9	29.1 ± 2.0 ^NS^	27.7 ± 2.4 ^0.008/0.002^
MCHC (g/dL)	32.0–35.0	33.1 ± 1.3	33.0 ± 1.2 ^NS^	32.5 ± 1.4 ^0.020/NS^
RDW (%)	11.5–14.5	13.9 ± 1.7	13.9 ± 0.9 ^NS^	14.3 ± 1.4 ^NS/0.046^
HbF (%)	0.5–1.3	0.41 ± 0.34	0.33 ± 0.13 ^NS^	0.40 ± 0.26 ^NS/NS^
HbA2 (%)	1.5–3.6	2.83 ± 0.34	2.81 ± 0.32 ^NS^	2.64 ± 0.35 ^0.005/0.009^
HbA0 (%)	>95.9	96.8 ± 0.5	96.9 ± 0.3 ^NS^	97.0 ± 0.5 ^0.027/NS^
p2 (related to HbA1C) (%)	<4.5	4.06 ± 0.26	4.79 ± 0.22 ^<0.001^	7.92 ± 1.87 ^<0.001/<0.001^

**Table 2:** Compartmentalization of Hb isoforms in healthy, prediabetic, and T2 diabetic individuals. The percentage of each Hb isoform in relation to total Hb is shown. Data are presented as median ± CI. Statistical significance was determined by Wilcoxon signed-rank test compared to the healthy group for prediabetic individuals and compared to healthy/prediabetic groups for T2 diabetic individuals. Significance level: *p* < 0.05; NS, non-significant.

	Healthy (*n* = 56)		Prediabetes (*n* = 53)		T2 Diabetes (*n* = 56)	
	Intact	Membrane	Intact	Membrane	Intact	Membrane
HbF	0.30 ± 0.9	0.30 ± 0.11	0.30 ± 0.04 ^NS^	0.30 ± 0.06 ^NS^	0.30 ± 0.07 ^NS/NS^	0.30 ± 0.08 ^NS/0.043^
HbA2	2.80 ± 0.09	4.50 ± 0.17	2.80 ± 0.09 ^NS^	4.30 ± 0.19 ^NS^	2.60 ± 0.09 ^0.001/0.003^	4.20 ± 0.24 ^NS/NS^
HbA0	96.8 ± 0.1	95.2 ± 0.2	96.8 ± 0.09 ^NS^	95.4 ± 0.2 ^NS^	97.0 ± 0.1 ^0.021/0.049^	95.2 ± 0.3 ^NS/NS^
HbA1C	5.33 ± 0.07	4.61 ± 0.08	6.00 ± 0.06 ^<0.001^	5.14 ± 0.08 ^<0.001^	8.58 ± 0.47 ^<0.001/<0.001^	6.91 ± 0.43 ^<0.001/<0.001^

**Table 3:** Pearson’s correlation coefficients for HbA1C vs. other Hb isoforms. The significance of the correlations (numerical values in parentheses below the coefficient) was accepted as *p* ≤ 0.05; NS, non-significant.

	Whole Cohort (*n* = 165)		Healthy (*n* = 56)		Prediabetes (*n* = 53)		T2 Diabetes (*n* = 56)	
	Intact	Membrane	Intact	Membrane	Intact	Membrane	Intact	Membrane
HbF	0.013 (NS)	−0.018 (NS)	0.146 (NS)	−0.011 (NS)	0.107 (NS)	−0.013 (NS)	−0.063 (NS)	−0.186 (NS)
HbA2	−0.334 (<0.001)	−0.318 (<0.001)	−0.263 (NS)	−0.525 (<0.001)	0.176 (NS)	−0.449 (<0.001)	−0.391 (0.003)	−0.630 (<0.001)
HbA0	+0.259 (<0.001)	+0.281 (<0.001)	0.082 (NS)	+0.415 (0.001)	−0.213 (NS)	+0.406 (0.003)	+0.342 (0.010)	+0.629 (<0.001)

**Table 4:** Pearson’s correlation coefficients for HbA1C vs. RBC functional markers. The correlations (numerical values in parentheses below the coefficient) were accepted as significant if *p* ≤ 0.05; NS, non-significant; n is number of independent tests.

	Healthy		Prediabetes		T2 Diabetes	
	Intact HbA1C	Membrane HbA1C	Intact HbA1C	Membrane HbA1C	Intact HbA1C	Membrane HbA1C
K^+^ loss (mM/h per Hb)	−0.040 (NS)	+0.043 (NS)	+0.010 (NS)	+0.009 (NS)	−0.037 (NS)	+0.166 (NS)
	(*n* = 15)		(*n* = 14)		(*n* = 14)	
Glucose consumption (mg/dL per hour per Hb)	+0.083 (NS)	−0.193 (NS)	+0.011 (NS)	−0.561 (0.037)	−0.222 (NS)	−0.206 (NS)
	(*n* = 15)		(*n* = 14)		(*n* = 14)	
Lactate release (mM/h per Hb)	−0.515 (0.049)	+0.469 (NS)	−0.489 (NS)	−0.034 (NS)	+0.318 (NS)	+0.264 (NS)
	(*n* = 15)		(*n* = 14)		(*n* = 14)	
Intact deprotonated reduced thiol (A.U., normalized)	+0.629 (0.012)	+0.403 (NS)	+0.497 (NS)	+0.252 (NS)	−0.144 (NS)	−0.284 (NS)
	(*n* = 15)		(*n* = 15)		(*n* = 17)	
Median elongation rate	+0.013 (NS)	+0.175 (NS)	−0.839 (0.005)	−0.256 (NS)	−0.345 (NS)	−0.222 (NS)
	(*n* = 9)		(*n* = 9)		(*n* = 9)	

## Data Availability

Data is contained within the article. The data presented in this study are available on request from the corresponding author.

## Supplementary Materials

The following supporting information can be downloaded at: https://www.mdpi.com/article/10.3390/ijms26209890/s1.
