# Supplementary material for: Hemoglobin A1C: Intracellular Heterogeneity and Functional Implications in Prediabetic and T2 Diabetic Erythrocytes
Source: Int J Mol Sci. 2025 Oct 11;26(20):9890. doi: 10.3390/ijms26209890 (PMC12564634; doi:10.3390/ijms26209890)

Supplementary Data

Table S1. Distribution of hemoglobin (Hb) isoforms in intact red blood cells (RBCs) and in their membrane and cytosolic compartments. Blood samples collected into heparin-supplemented tubes were kept at room temperature prior to the experimental manipulations. Total time elapsed between blood collection and measurement did not exceed 4 h. RBCs were isolated from plasma and buffy coat by short centrifugation at 1700*g*, and measurement of Hb isoforms in intact RBCs was immediately performed. Then, RBCs were lysed with ice-cold HEPES-based hypoosmotic solution, and hemolysates were electrophoresed to evaluate fractions of each Hb isoform in the RBC cytosol; the procedure was repeated three more times to obtain membranes for determination of Hb isoform distribution. Percent of each Hb isoform out of total Hb is shown. Data are presented as means ± SD. Significance (presented as superscript values) was determined compared to corresponding intact RBC datasets using paired Student's t-test. Significance level: $p \leq 0.05$; NS, non-significant.

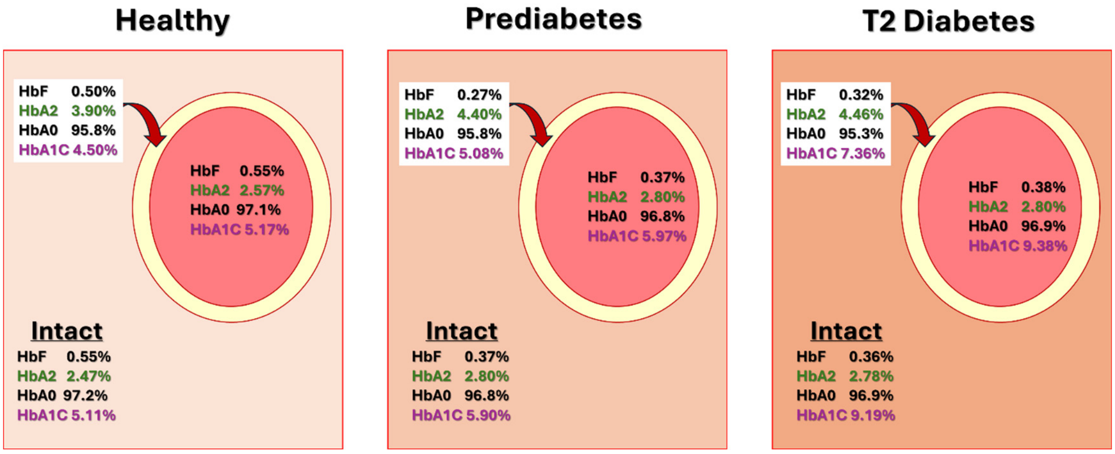

	**Healthy (n = 3)**			**Prediabetes (n = 3)**			**Type 2 (T2) Diabetes (n = 5)**		
	Intact	Membrane	Cytosol	Intact	Membrane	Cytosol	Intact	Membrane	Cytosol
HbF	0.55±0.07	0.50±0.00^{NS}	0.55±0.07 NS	0.37±0.06	0.27±0.23^{NS}	0.37±0.06 NS	0.36±0.11	0.32±0.08^{NS}	0.38±0.13 NS
HbA2	2.47±0.31	3.90±0.17$^{0.004}$	2.57±0.31 NS	2.80±0.17	4.40±0.36$^{0.024}$	2.80±0.10 NS	2.78±0.42	4.46±0.84$^{0.003}$	2.80±0.41 NS
HbA0	97.2±0.5	95.8±0.4$^{0.007}$	97.1±0.5 NS	96.8±0.2	95.8±0.4$^{0.010}$	96.8±0.2 NS	96.9±0.4	95.3±0.2$^{0.003}$	96.9±0.3^{NS}
HbA1C	5.11±0.33	4.50±0.24$^{0.011}$	5.17±0.29 NS	5.90±0.25	5.08±0.34$^{0.019}$	5.97±0.20 NS	9.19±2.01	7.36±1.23$^{0.007}$	9.38±1.97^{NS}

Table S2. Mean content and concentrations of Hb isoforms in intact RBCs and their membranes from healthy, prediabetic, and T2 diabetic individuals. Membrane content values were determined by combining data on the relative fraction of each Hb isoform out of total Hb, along with measurements of total mean corpuscular hemoglobin (MCH) and its concentration (MCHC) for intact RBCs, and total Hb concentrations in RBC membranes obtained via Drabkin's method. All datasets exhibited a normal distribution and were statistically compared using paired Student's t-tests. Significance level: $p < 0.05$; NS, non-significant.

	**Healthy (n = 12)**			**Prediabetes (n = 12)**			**T2 Diabetes (n = 11)**		
	Intact	Intact	Membrane	Intact	Intact	Membrane	Intact	Intact	Membrane
	MCH (pg)	MCHC (g/L)	MCHC (g/L)	MCH (pg)	MCHC (g/L)	MCHC (g/L)	MCH (pg)	MCHC (g/L)	Membrane content (g per g total normal Hb)
HbF	0.09+0.07	0.11+0.08	0.004+0.002	0.09+0.07 ^{NS}	0.11+0.04 ^{NS}	0.004+0.001 ^{NS}	0.10+0.06 $^{NS/NS}$	0.11+0.07 $^{NS/NS}$	0.004+0.002 $^{NS/NS}$
HbA2	0.82+0.07	0.94+0.06	0.046+0.006	0.79+0.13 ^{NS}	0.91+0.12 ^{NS}	0.044+0.008 ^{NS}	0.67+0.10 $^{<0.001/0.022}$	0.79+0.10 $^{<0.001/0.014}$	0.043+0.005 $^{NS/NS}$
HbA0	28.2+1.6	32.2+1.3	1.032+0.067	27.8+1.8 ^{NS}	32.3+1.3 ^{NS}	1.090+0.108 ^{NS}	26.7+2.2 $^{NS/NS}$	31.5+1.2 $^{NS/NS}$	1.022+0.078 $^{NS/NS}$
HbA1C	1.19+0.09	1.36+0.08	0.036+0.004	1.38+0.11 $^{<0.001}$	1.60+0.10 $^{<0.001}$	0.045+0.004 $^{<0.001}$	2.06+0.54 $^{<0.001/0.002}$	2.44+0.61 $^{<0.001}$	0.063+0.020 $^{<0.001/0.011}$

Table S3. Alterations in the near-membrane distribution of Hb isoforms following 4-h incubation of erythrocytes in a hyperglycemic environment (10 mM D-glucose in plasma-mimicking buffer [PMB]). Percentage of each Hb isoform relative to total Hb is presented as median ± CI. Statistical significance of compared paired RBC membrane datasets (pre- and post-incubation) for each experimental group was tested by Wilcoxon signed-rank test. NS indicates non-significant changes.

	**Healthy (n = 18)**			**Prediabetes (n = 17)**			**T2 Diabetes (n = 17)**		
	Intact	Membrane 0 h	Membrane 4 h	Intact	Membrane 0 h	Membrane 4 h	Intact	Membrane 0 h	Membrane 4 h
HbF	0.30±0.05	0.30±0.06	0.30±0.06 NS	0.30±0.05	0.30±0.05	0.30±0.14 NS	0.50±0.10	0.50±0.16	0.50±0.16 NS
HbA2	2.90±0.08	3.80±0.31	4.00±0.38 NS	2.70±0.12	4.20±0.31	4.10±0.34 NS	2.70±0.13	3.60±0.34	3.70±0.36 NS
HbA0	96.9±0.08	96.0±0.3	95.7±0.4 NS	97.1±0.1	95.4±0.3	95.7±0.4 NS	97.0±0.1	95.8±0.4	95.9±0.4 NS
HbA1C	5.43±0.10	4.66±0.16	4.66±0.22 NS	6.09±0.54	5.23±0.57	5.23±0.53 NS	9.15±0.87	7.81±0.83	7.81±0.76 NS

Table S4. Effect of EDTA on Hb isoform distribution in RBC membranes. Percent of each Hb variant out of total Hb is shown. Percent of each Hb variant out of total Hb is shown. Data are presented as median ± CI. Significance was determined compared to corresponding RBC membrane datasets using Wilcoxon signed-rank test at $p \leq 0.05$; NS, non-significant. For more details, see legend to Figure 3.

	**Healthy (n = 10)**			**Prediabetes (n = 9)**			**T2 Diabetes (n = 8)**		
	Intact	Membrane	Membrane + 5 mM EDTA	Intact	Membrane	Membrane + 5 mM EDTA	Intact	Membrane	Membrane + 5 mM EDTA
HbF	0.40±0.11	0.50±0.13	0.40±0.19 ^{NS}	0.30±0.06	0.30±0.16	0.30±0.13 ^{NS}	0.25±0.12	0.30±0.29	0.45±0.20 ^{NS}
HbA2	2.90±0.23	4.95±0.36	9.85±1.02$^{<0.001}$	2.80±0.24	4.20±0.40	7.90±1.12$^{<0.001}$	3.05±0.30	4.55±0.73	9.55±1.77$^{0.002}$
HbA0	96.7±0.3	94.6±0.36	89.9±1.0$^{<0.001}$	96.8±0.24	95.4±0.5	91.7±1.1$^{<0.001}$	96.8±0.3	95.2±0.6	90.0±1.7$^{0.002}$
HbA1C	5.23±0.12	4.52±0.16	3.42±0.38 $^{0.001}$	6.00±0.13	5.23±0.14	3.90±0.16$^{<0.001}$	7.40±0.64	7.05±0.54	4.95±0.37$^{<0.001}$

Table S5. RBC functional markers in healthy, prediabetic, and T2 diabetic individuals. Data for K^+ loss, glucose consumption, and lactate release are presented as means of n independent tests ± SD. Statistical significance for these tests was determined by Student's t-test compared to the healthy group for prediabetic individuals and compared to healthy/prediabetic groups for T2 diabetic individuals. Data for intact deprotonated reduced thiol and median elongation rate are presented as median ± CI, and significance was determined using Wilcoxon signed-rank test at $p \leq 0.05$.

	**Healthy**	**Prediabetes**	**T2 Diabetes**
K^+ loss (mM/h per Hb)	0.020±0.007 (n=15)	0.025±0.010 ^{NS} (n=14)	0.026± 0.010 $^{NS/NS}$ (n=14)
Glucose consumption (mg/dL per h per Hb)	0.629±0.130 (n=15)	0.412± 0.178$^{0.001}$ (n=14)	0.476± 0.134 $^{0.004/NS}$ (n=14)
Lactate release (mM/h per Hb)	0.053±0.010 (n=15)	0.056±0.006 ^{NS} (n=14)	0.058± 0.007 $^{NS/NS}$ (n=14)
Intact deprotonated reduced thiol (A.U., normalized)	1.038±0.058 (n=15)	0.992±0.062 ^{NS} (n=15)	0.916±0.088 $^{NS/NS}$ (n=17)
Median elongation rate	1.47±0.05 (n=9)	1.46±0.03 ^{NS} (n=9)	1.47±0.05 $^{NS/NS}$ (n=9)

Figure S1. Minimal changes in membrane Hb content after a 4-h incubation in PMB supplemented with 10 mM glucose. Hb content was quantified using Drabkin's reagent. Significance was assessed by paired Student's t-test comparing 0-h and 4-h values - NS, not significant). Hb concentration in intact RBCs from healthy, prediabetic, and overt T2 diabetic individuals were 336.7 ± 7.4, 346.8 ± 9.8 and 333.5 ± 7.9 g/l, respectively.

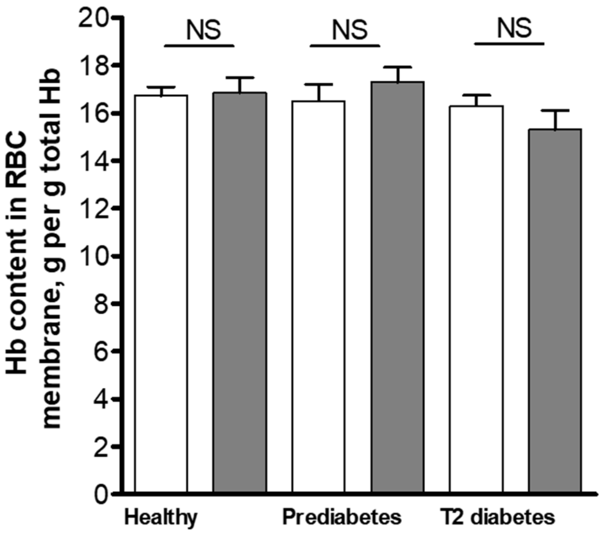

Supplement: Supplementary file 1 [file ijms-26-09890-s001.zip › ijms-3898041-supplementary.pdf]
